# Supplementary material for: Whey Protein Hydrolysate Exerts Anti-Inflammatory Effects to Alleviate Dextran Sodium Sulfate (DSS)-Induced Colitis via Microbiome Restoration
Source: Nutrients. 2023 Oct 17;15(20):4393. doi: 10.3390/nu15204393 (PMC10610201; doi:10.3390/nu15204393)
Supplement: Supplementary file 1 [file nutrients-15-04393-s001.zip › nutrients-2659100-supplementary.pdf]

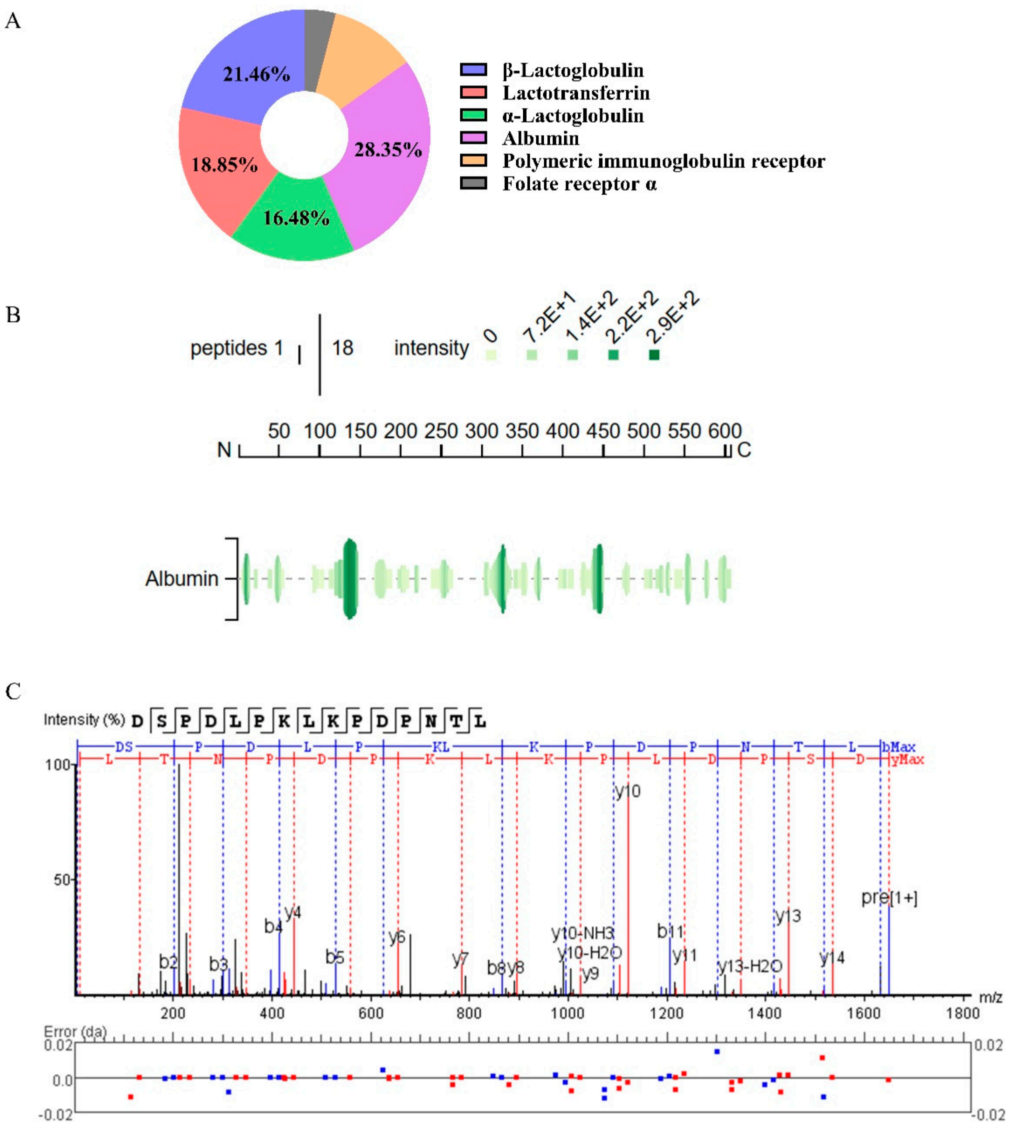

Figure S1. The composition of WPH. (A) The proportion of parent protein for WPH. (B) The peptide profile of the most abundant albumin in WPH. (C) The best unique peptide-spectrum matches (PSM).

Table S1. Disease Activity Index score

Score	Weight loss rate（%）	Stool properties	Fecal occult blood
0	0	Normal	Negative
1	1-5	Weak soft stool	Weak positive
2	6-10	Soft stool	Positive
3	11-15	Mucus-like stool	Strong positive
4	>15	Watery stool	Blood fecal stool
